# Bone disease in kidney transplant: don’t forget about osteomalacia: a case report and literature review

**DOI:** 10.1007/s11255-025-04781-y

**Published:** 2025-09-25

**Authors:** Francesco Aguanno, Alessia Passaseo, Simona Barbuto, Daniele Vetrano, Guido Zavatta, Guido Marzocchi, Sandro Giannini, Giorgia Comai, Gaetano La Manna, Giuseppe Cianciolo

**Affiliations:** 1 Department of Medical and Surgical Sciences (DIMEC), Alma Mater Studiorum University of Bologna, Bologna, Italy; 2 Nephrology, Dialysis and Kidney Transplant Unit, IRCCS Azienda Ospedaliero-Universitaria di Bologna, Bologna, Italy; 3 Division of Endocrinology and Diabetes Prevention and Care, IRCCS Azienda Ospedaliero-Universitaria di Bologna, Bologna, Italy; 4 Pediatric and Adult CardioThoracic and Vascular, Oncohematologic and Emergency Radiology Unit, IRCCS Azienda Ospedaliero-Universitaria di Bologna, Bologna, Italy; 5 Department of Medicine, Clinica Medica I, University of Padova, Padua, Italy

**Keywords:** Osteomalacia, Kidney transplantation, Vitamin D deficiency, Hypophosphatemia, Secondary hyperparathyroidism, Case report, Literature review

## Abstract

**Introduction:**

Osteomalacia is an often-overlooked manifestation of post-transplant bone disease that may persist or newly develop in kidney transplant recipients because of pre-existing chronic kidney disease–mineral and bone disorder, ongoing immunosuppression, and alterations in calcium-phosphate metabolism. Severe vitamin D deficiency, hypophosphatemia, and secondary hyperparathyroidism create a metabolic milieu that favors osteoid mineralization defect and leads to debilitating skeletal pain and fragility fractures.

**Objective:**

This case report documents the clinical course, diagnostic work-up, and therapeutic response of a kidney-transplant recipient with severe vitamin D deficiency, with the aim of raising awareness of this condition and outlining practical management strategies.

**Case report:**

A 61-year-old woman underwent living-donor kidney transplantation in 2020. Four months later, she presented with diffuse bone pain, progressive gait impairment, and laboratory evidence of hypercalcemic hyperparathyroidism (PTH 130 pg/mL), severe vitamin D deficiency (25[OH]D 7 ng/mL), and hypophosphatemia (2.8 mg/dL). Very high levels of bone-specific alkaline phosphatase may reflect both bone mineralization defect and high bone turnover. Imaging supported the diagnosis of osteomalacia, revealing bone-marrow edema of both knees, Looser zones, and focal radiotracer uptake on ^99mTc-MDP scintigraphy. The patient started treatment with high-dose cholecalciferol (60,000 IU/day) followed by monthly calcifediol, together with continued cinacalcet and subsequent oral bisphosphonate therapy; this regimen normalized 25(OH)D (42 ng/mL), reduced bone-turnover markers, and enabled the recovery of independent ambulation within 9 months. Follow-up dual-energy X-ray absorptiometry showed lumbar BMD improvement (T-score −3.7 to −2.6) and stabilization of femoral osteopenia at 26 months post-transplant.

**Conclusion:**

Early recognition of osteomalacia after kidney transplantation and aggressive correction of vitamin D deficiency, phosphate wasting, and hyperparathyroidism can result in rapid symptomatic relief and partial reversal of bone loss. Routine monitoring of mineral metabolism and bone turnover markers should therefore be integrated into post-transplant care to prevent delayed diagnosis. Controlled studies are warranted to define optimal supplementation protocols and thresholds in this population.

## Introduction

Kidney transplantation remains the optimal renal replacement therapy for eligible dialysis-dependent patients with kidney failure, as it fully restores kidney function. Compared to patients maintained on dialysis, kidney transplant recipients (KTRs) exhibit improved survival, reduced dialysis-associated morbidity, lower cardiovascular risk, enhanced quality of life, and reduced healthcare costs [1, 2]. However, bone and mineral disorders are still a burden in KTRs [3, 4]. Various clinical studies have shown ongoing disturbances of mineral metabolism and a progressive loss of bone mass after kidney transplantation, especially during the first post-transplant year, as evidenced by laboratory parameters and imaging techniques [3–9]. In particular, as kidney function begins to recover, several alterations in calcium-phosphorus metabolism occur, including hypophosphatemia, hypercalcemia, hyperparathyroidism, and vitamin D deficiency [10–13]. In this context, fibroblast growth factor 23 (FGF23) plays an important role in phosphate and vitamin D metabolism and remains elevated in the immediate post-transplant period, reflecting the patient’s pre-transplant mineral metabolism abnormalities. However, as graft function stabilizes and phosphate homeostasis improves, FGF23 concentration declines, typically around three months after transplantation. By one year post-transplant, FGF23 levels continue to decrease and usually reach a new steady state that corresponds to the patient’s level of kidney function. At this point, FGF23 concentration is often comparable to that observed in non-transplanted chronic kidney disease (CKD) patients with similar estimated glomerular filtration rate (eGFR) values, suggesting that kidney function, rather than transplant status per se, becomes the primary determinant of FGF23 levels [14, 15].

Although it has long been assumed that successful kidney transplantation largely resolves CKD–mineral and bone disorder (CKD-MBD), abnormalities of mineral and bone metabolism can still present in KTRs. This condition is known as post-transplantation bone disease (PTBD) and is characterized by a bone phenotype that reflects all the patient’s pre-transplant complications, even before CKD onset.

Hypophosphatemia, hypercalcemia, and hypovitaminosis D are indeed highly prevalent among KTRs [12]. However, secondary hyperparathyroidism (SHPT) completely resolves in only 30% and 57% of transplanted patients within the first and second year post-transplantation, respectively.

Post-transplant hyperparathyroidism can be distinguished into a persistent (maladaptive response) versus de novo form (compensatory adaptive response). Persistent hyperparathyroidism results from preexisting CKD-MBD with SHPT, while de novo hyperparathyroidism is predominantly elicited by decreasing graft function. The main predictive factors of persistent SHPT after transplant are dialysis vintage, high pre-transplant parathyroid hormone (PTH) levels, and the size of the parathyroid glands [15]. In KTRs, these changes are largely attributable to pre-existing bone damage and CKD–MBD that persist after transplantation, de novo CKD–MBD, and the effects of immunosuppressive therapy, particularly corticosteroids and calcineurin inhibitors. The contribution of each component to the overall scenario changes over time [12, 13, 16–19].

Vitamin D deficiency, defined as 25(OH)D levels <30 ng/mL, is very common following transplantation, occurring in up to 80% of KTRs by three months post-transplantation and persisting in the short- and long-term even in the presence of normal graft function [4, 12].

Osteomalacia is a metabolic bone disease characterized by an impaired mineralization of newly formed osteoid due to vitamin D deficiency and/or insufficient calcium and phosphate availability that leads to hypocalcemia, hypophosphatemia, and bone loss. Reduced availability of vitamin D due to poor production and/or absorption of cholecalciferol induces deficiency of the physiologically active form of 1,25(OH)_2_D resulting in defective mineralization of osteoid. Bone formation is driven by the deposition of hydroxyapatite crystals on the osteoid matrix. These changes may also reflect an apparent resistance of bone cells to vitamin D, resulting in an impaired response of vitamin D receptors (VDR) [4]. Patients with advanced kidney disease are at increased risk for a spectrum of bone disorders, including osteitis fibrosa, adynamic bone disease, and osteomalacia. Osteomalacia is particularly frequent among KTRs, yet it often remains underdiagnosed in both pre- and post-transplant settings [19, 20]. Notably, bone biopsy studies in KTRs have revealed a significant prevalence of focal or generalized osteomalacia, even in patients with normal calcitriol levels [20].

Hypophosphatemia is frequently observed in the first months after kidney transplantation and is usually multifactorial, transient, and asymptomatic. Persistent hypophosphatemia resulting from excessive kidney phosphorus wasting is an important but overlooked cause of osteodystrophy in the kidney transplant population [7, 20].

This report describes the case of a patient with severe osteoarticular pains secondary to severe vitamin D deficiency, hypophosphatemia, persistent hyperparathyroidism, and BMD loss after successful kidney transplantation.

## Case report

A 61-year-old woman underwent kidney transplantation from a related living donor. She had a history of CKD secondary to IgA nephropathy, diagnosed in 1997, for which she was initially treated with peritoneal dialysis for 70 months, followed by hemodialysis for approximately eight months beginning in 2020.

During the dialysis period, the patient developed SHPT, which was managed with cinacalcet, calcitriol, and paricalcitol, in accordance with the KDIGO guidelines [21]. However, these treatments were not effective, as proven by persistently elevated PTH levels of up to 690 pg/mL (normal range: 12–88 pg/mL), hyperphosphatemia (serum phosphate 7.7 mg/dL), and a mild tendency toward hypercalcemia (serum calcium 10 mg/dL). Imaging studies revealed enlarged parathyroid glands. Two months prior to the kidney transplant, the patient underwent selective right inferior parathyroidectomy, which histologically confirmed the presence of parathyroid adenoma.

In 2020, the patient received a kidney transplant from a living donor, with compatible blood group and an identical HLA panel. Induction therapy with basiliximab was not administered, as the transplant was from a living donor with identical HLA matching. The patient was started on a triple immunosuppressive regimen consisting of corticosteroids, tacrolimus (which was switched to cyclosporine after two months), and mycophenolic acid for maintenance therapy.

**Fig. 1:**
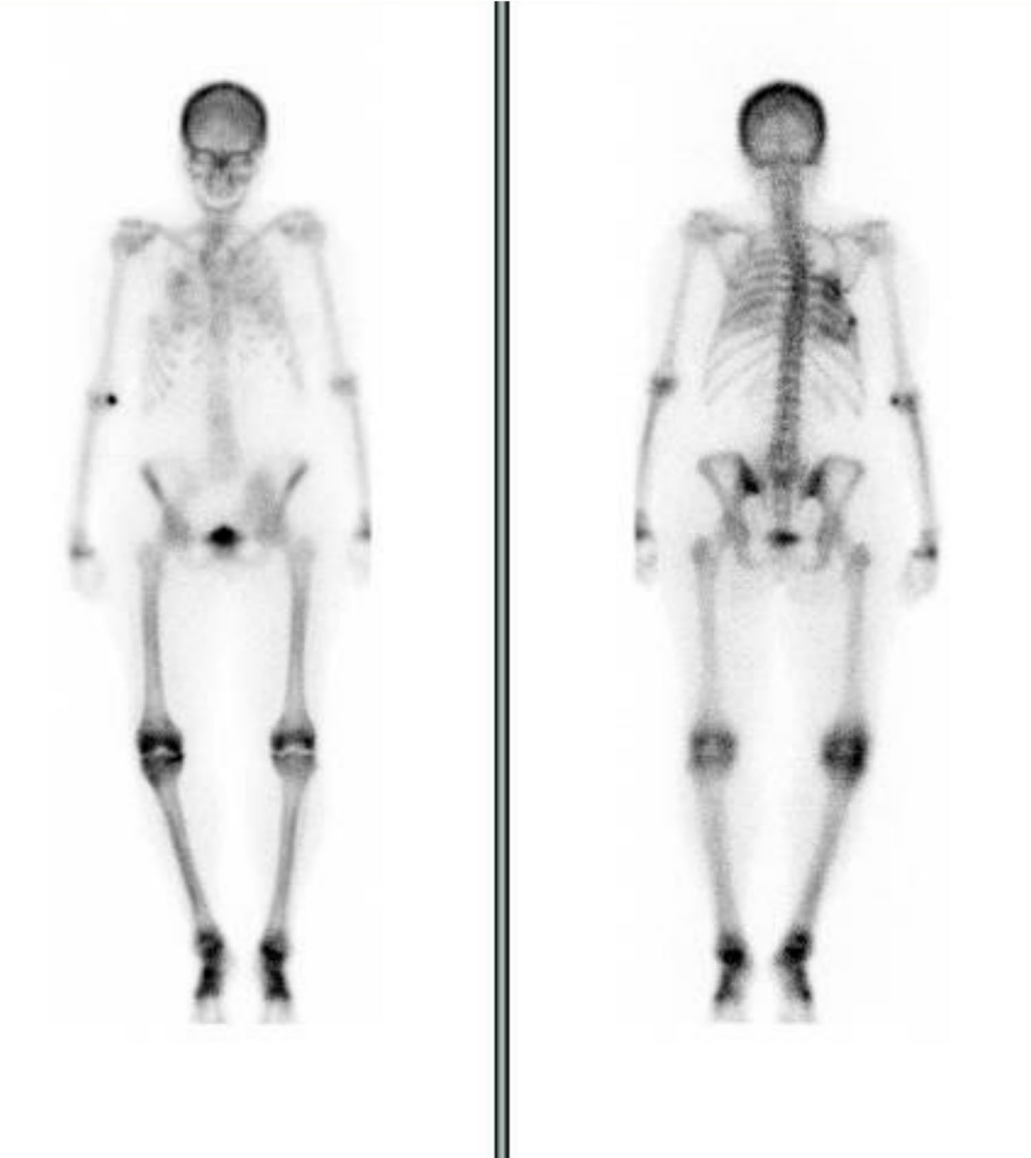
Skeletal scintigraphy 99m Tc-MDP

The patient presented to our clinic approximately four months after kidney transplant with a recurrence of hypercalcemic hyperparathyroidism (calcium 10.7 mg/dL; PTH 130 pg/mL). She complained of widespread joint pain, particularly in the lower limbs, without signs of inflammation, and reported progressively worsening difficulty with ambulation. Blood tests showed a satisfying graft function with creatinine at 1.1 mg/dL and mildly reduced eGFR (54 mL/min/1.73 m^2^), hypercalcemia (calcium 11.2 mg/dL), hypophosphatemia (phosphate 2.8 mg/dL), increased bone formation and resorption markers (osteocalcin 116.6 ng/mL; bone alkaline phosphatase ([BALP] 105 µg/L, crosslaps 4082 ng/mL). BALP was markedly higher than osteocalcin, a pattern that is consistent with a mineralization defect and supportive of the diagnosis of osteomalacia. PTH decreased to 95 pg/mL, thus approaching the normal range, while the patient was receiving cinacalcet at a dose of 30 mg/day. Additionally, severe vitamin D deficiency was noted, with a level of 7 ng/mL. The FePI (Fractional Excretion of Phosphate) was 30.6%, indicating renal phosphate wasting. Skeletal scintigraphy 99m Tc-MDP revealed focal areas of radiotracer hyperaccumulation in proximity to the lateral wall of the 4th right rib, right elbow, left wrist, bilateral knees and ankles (Fig. 1).

Vertebral morphometry showed two fractures of the vertebral bodies of D6 and D7 and reduced L5-S1 disk space. Dual-Energy X-ray Absorptiometry (DEXA) scan showed lumbar osteoporosis (L1-L4 T score −3.7) and femoral osteoporosis (T score −2.9). A contrast-enhanced ultrasound of the parathyroids identified an enlarged, hyperfunctioning parathyroid (measuring 11 × 3 mm).

**Fig. 2:**
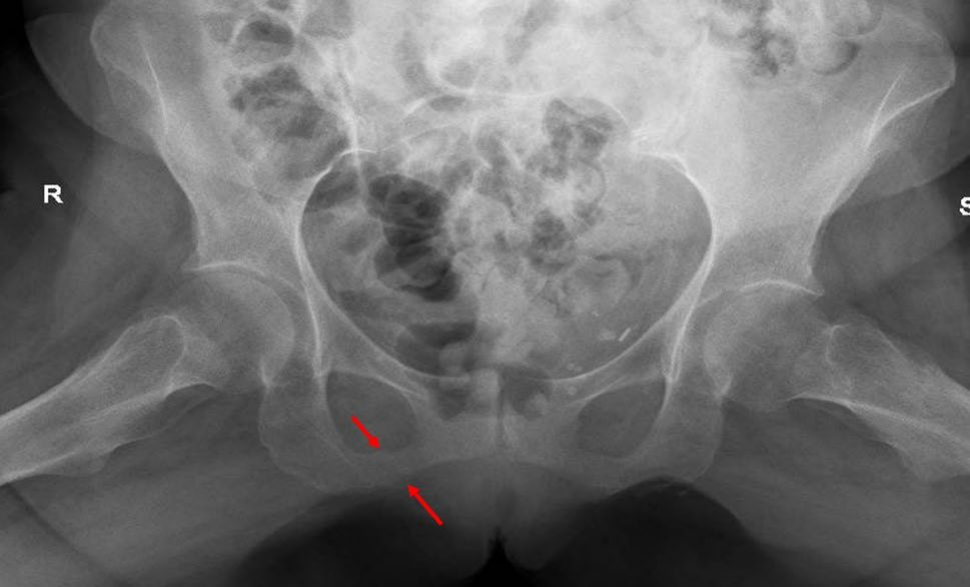
X-rays of the shoulders and right knee

X-rays of the shoulders and right knee were negative for fractures. Magnetic resonance imaging (MRI) of the left knee revealed diffuse trabecular edema in the patella, tibial plateau, and femoral condyles. An area of bone rarefaction compatible with Looser zones was identified on the hip X-ray (Fig. 2).

Based on these findings, we suspected osteomalacia secondary to severe vitamin D deficiency and started the administration of therapy with cholecalciferol at a dose of 60,000 IU per day.

**Fig. 3:**
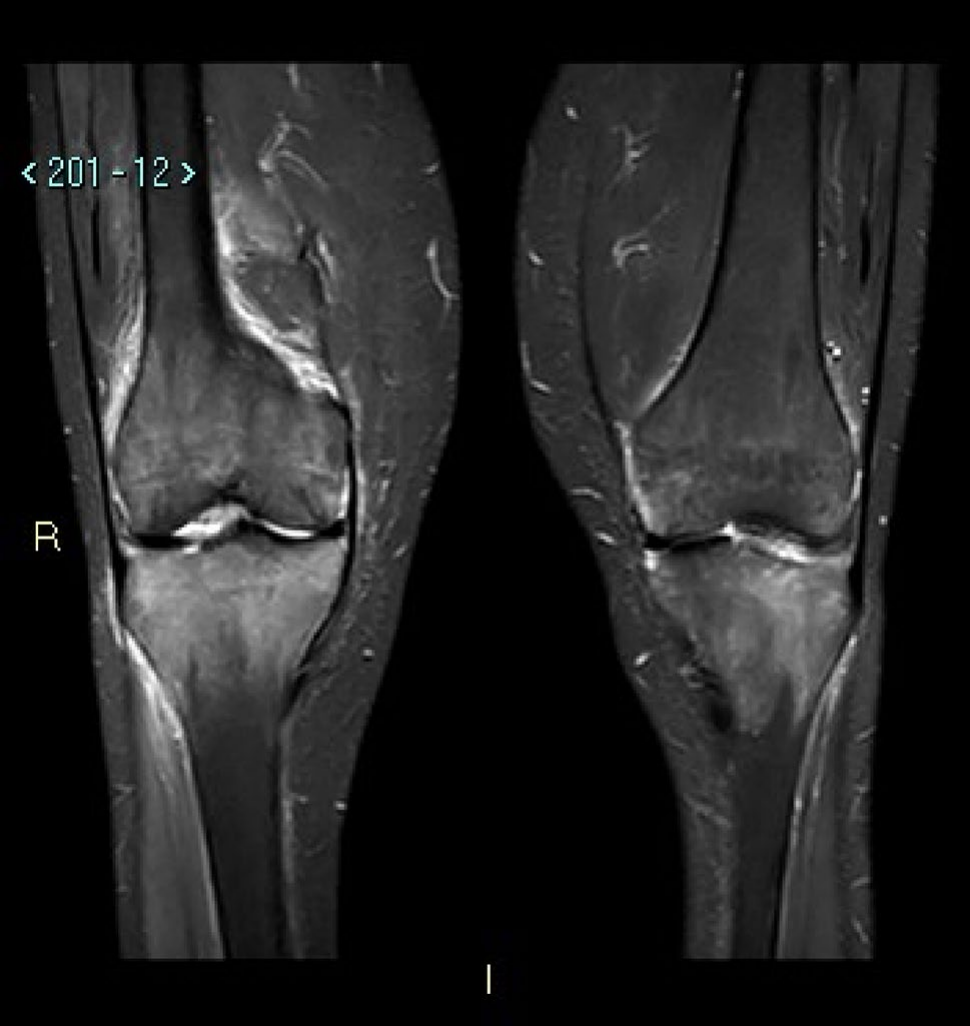
Bilateral knee MRI, six months post-transplant

One month later (six months post-transplant), the patient came to our outpatient clinic in a wheelchair due to persistent walking impairment, although she reported partial relief of pain. Bilateral knee MRI (Fig. 3) highlighted significant bone marrow edema, while laboratory tests showed only a modest increase in serum 25(OH)D levels (19 ng/mL), with persistently elevated bone turnover markers. As a result, calcifediol (0.266 mg, one tablet per month) was added to the treatment regimen, without introducing antiresorptive therapy.

**Fig. 4:**
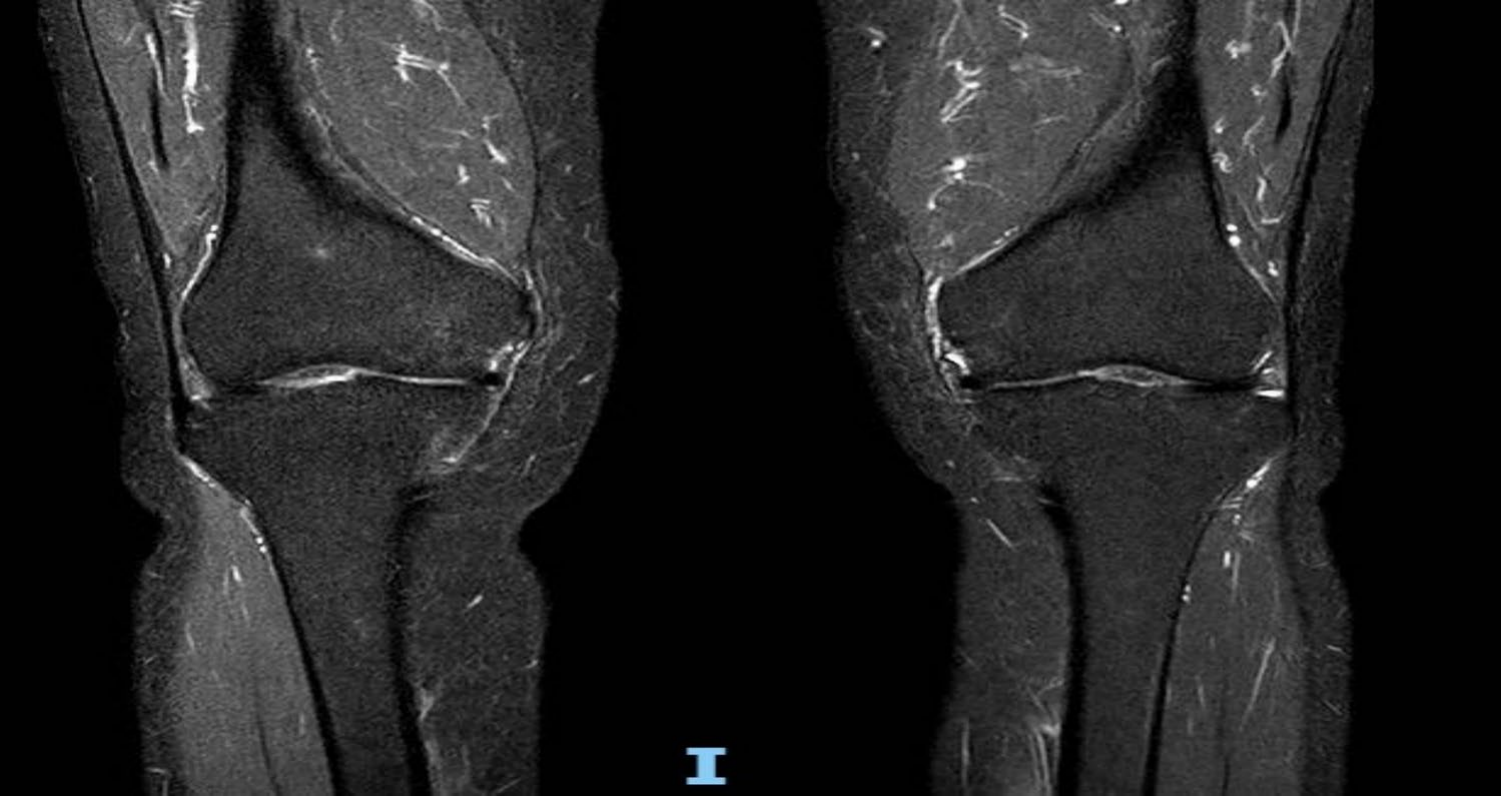
Bilateral knee MRI, nine months post-transplant and five months of native vitamin D supplementation initiation

After five months of native vitamin D supplementation and nine months post-transplant, the patient was able to walk independently with the aid of crutches, experiencing significant pain relief and achieving a 25(OH)D level of 42 ng/mL. A follow-up bilateral knee MRI showed a reduction in bone marrow edema (Fig. 4).

We recommended antiresorptive therapy with oral bisphosphonates; however, the patient did not begin treatment due to the need for dental implants.

At 26 months post-kidney transplant, the patient was fully independent in ambulation without requiring assistive devices.

**Fig. 5:**
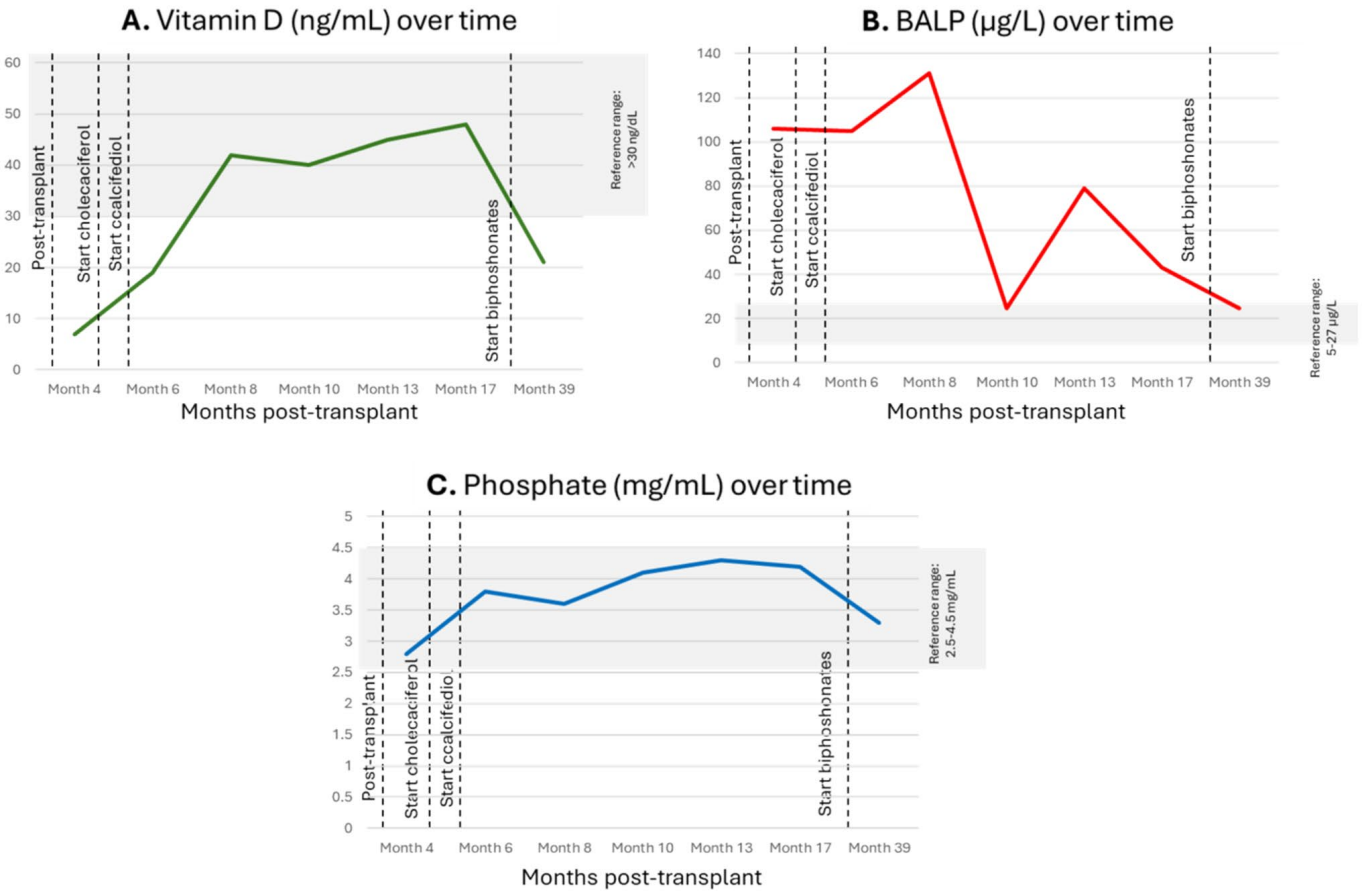
Changes over time of: **A** vitamin D; **B** BALP; and **C** phosphate

As detailed in Table 1, laboratory tests showed persistent SHPT with a PTH level of 132 pg/mL, a trend toward hypercalcemia (serum calcium 10.8 mg/dL), and increased bone turnover markers, though with improvements compared to previous evaluations (osteocalcin 47.2 ng/mL, BALP 24.8 µg/L, and serum C-terminal telopeptide of type I collagen [CTX] 0.963 ng/mL). The trends over time of vitamin D, BALP, and phosphate are detailed in Fig. 5 A, B and C, respectively.

**Table 1:** Serum laboratory parameter trends over time (months post-transplant)

	Reference range	Month 4	Month 6	Month 8	Month 10	Month 13	Month 17	Month 39
Creatinine^a^, mg/dL	0.6–1.1	1.10	1.06	0.92	0.92	1.06	1.18	0.82
Calcium, mg/dL	8.8–10.4	11.2	10.9	10.6	10.7	10.2	9.9	10.8
Phosphate, mg/dL	2.5–4.5	2.8	3.8	3.6	4.1	4.3	4.2	3.3
PTH^b^, pg/mL	12–88	94	82	102	148	135	118	132
Osteocalcin, mg/dL	10–46	116.5	140.6	182.4	254	254	102.8	47.2
BALP, μg/L	5–27	106	105	131	24.8	79	43	24.8
Cross-laps, ng/mL	0.112–0.738	4.082	4.018	5.036	4.939	1.988	1.506	0.963
Vitamin D, ng/L	>30	7	19	42	40	45	48	21

*BALP* bone alkaline phosphatase, *PTH* parathyroid hormone

^a^Serum creatinine reference range is given for female sex

^b^Second generation PTH assay

DEXA revealed persistent lumbar osteoporosis with an improved T-score (L1–L4 BMD 0.817 g/cm^2^, T-score −3.0) and femoral osteopenia (femoral neck BMD 0.710 g/cm^2^, T-score −2.3; total femur BMD 0.821 g/cm^2^, T-score −1.5) (Table 2).

**Table 2:** Changes in bone mineral density

	Month 6	Month 25	Month 39
L1–L4			
T-score	−3.7	−2.8	−3
aBMD	0.738	0.842	0.817
Total hip			
T-score	−2.8	−2.3	−1.5
aBMD	0.661	0.767	0.821
Femoral neck			
T-score	−2.9	−2	−2.3
aBMD	0.628	0.737	0.710

Notes: T-score: T-score < −2.5 = Osteoporosis, T-score between −1 and −2.5 = Osteopenia; aBMD: Density — bone mineral density measured per square centimeters (g/cm^2^); L1–L4: lumbar vertebrae 1–4: Total hip: total hip region

*aBMD* areal bone mineral density, *BMD* bone mineral density

Vertebral morphometry revealed stable anterior wedge deformities at T6 (5 mm reduction in anterior vertebral height) and T7 (7 mm reduction). Based on these findings, we again recommended initiating antiresorptive therapy with bisphosphonates, which the patient agreed to start.

After one year of bisphosphonate therapy, she presented in good overall clinical condition and stable kidney function within normal limits. However, hypercalcemic hyperparathyroidism persisted, and bone turnover markers remained mildly elevated but showed further improvement (osteocalcin 62.5 ng/mL, BALP 25 µg/L, and serum C-terminal telopeptide of type I collagen [CTX] 0.677 ng/mL). Notably, BALP was disproportionately elevated compared to osteocalcin.

DEXA showed improvement in lumbar osteoporosis (L1–L4 BMD 0.863 g/cm^2^, T-score −2.6), while femoral osteopenia remained stable (femoral neck BMD 0.712 g/cm^2^, T-score −2.2; total femur BMD 0.809 g/cm^2^, T-score −1.6). X-ray imaging showed regression of bone rarefaction consistent with Looser zones.

## Discussion and literature review

PTBD often presents a more complex clinical and pathophysiological scenario than the alterations associated with CKD-MBD. In de novo KTRs treated with a steroid minimization immunosuppressive protocol, BMD changes especially in the first post-transplant year are highly variable, with 30–40% of patients showing substantial bone loss. Decreasing levels of PTH and bone turnover markers within the first 3 months post-transplant are associated with reduced BMD loss, and in some cases even BMD gain. Conversely, persistently elevated bone resorption markers throughout the first post-transplant year are linked to greater BMD loss. Delayed mineralization is present in KTRs in a variable range between 22 and 88%. This variability is related to the definition used in the studies to describe abnormal mineralization and with no clear trends in mineralization depending on the time since transplantation [22–25].

Although in KTRs, osteomalacia often results from vitamin D deficiency pre-existing to the kidney transplant, immunosuppression may influence the vitamin D metabolism [26]. Jorgensen et al. reported that commonly used immunosuppressive agents can adversely affect bone metabolism [25]. Glucocorticoids enhance the activity of 24-hydroxylase; this leads to reduced levels of 1,25-dihydroxyvitamin D. Additionally, glucocorticoids may downregulate VDR expression, thereby inducing a state of vitamin D resistance [4, 16, 25, 27].

Calcineurin inhibitors, such as cyclosporine A and tacrolimus, impair vitamin D metabolism by suppressing VDR expression in the kidney, leading to decreased expression of vitamin D-dependent calcium-binding proteins [19]. This suppression results in kidney calcium wasting despite elevated plasma levels of 1,25(OH)_2_D, suggesting a state of vitamin D resistance. Furthermore, both calcineurin inhibitors and 1,25(OH)_2_D are substrates of the cytochrome P450 enzyme CYP3A4. Collectively, these alterations contribute to an increased risk of bone disease and underscore the importance of tailored vitamin D supplementation in immunosuppressed populations [28].

Vitamin D deficiency results in hypocalcemia, hypophosphatemia, bone loss, deranged bone turnover, and mineralization. Osteomalacia is characterized by the accumulation of unmineralized osteoid, leading to bone softening and reduced strength. In severe cases, mineralization may halt completely, but lesions can reverse with correction of metabolic abnormalities [23, 24]. Hypophosphatemia and vitamin D deficiency often coexist in KTRs, and both contribute to osteomalacia: adequate PO4 and vitamin D levels are necessary for normal biomineralization [29]. In KTRs, histomorphometry findings often show increased osteoid volume and a higher likelihood of delayed mineralization as compared with patients with normal phosphate levels [22, 26].

Hypophosphatemia occurs in the early months following kidney transplantation, often due to persistent hyperparathyroidism and elevated FGF23 levels, which increase urinary phosphate excretion [3]. These abnormalities usually resolve within one year, but can contribute to osteomalacia by reducing phosphate availability for bone mineralization. FGF23 also suppresses 1α-hydroxylase and induces 24-hydroxylase, leading to lower active vitamin D levels, while PTH exerts the opposite effect. In our patient, hypophosphatemia related to high PTH, vitamin D deficiency, probable excess FGF23, likely amplified the metabolic milieu for osteomalacia [14, 15].

Although vitamin D deficiency is common in KTRs and can contribute to osteomalacia, it is often overlooked if hypercalcemia is present, and in this circumstance its supplementation is avoided. Moreover, persistence of this deficiency is clinically relevant since the coexistence with hyperparathyroidism may worsen skeletal fragility despite normalized kidney function [23, 25, 27].

Hypercalcemic hyperparathyroidism and widespread bone and joint pain (four months after kidney transplant) are the causes for patient referral to our clinic dedicated to bone and mineral disease. The coexistence of hypercalcemia and hyperparathyroidism is considered a distinct though less frequent (21.5%) phenotype of persistent hyperparathyroidism, referred to as tertiary hyperparathyroidism [25]. Moreover, it must be emphasized that hypercalcemic hyperparathyroidism may not necessarily be an expression of high skeletal remodeling but may be related to increased renal calcium reabsorption (lower levels of calciuria): the mechanisms underlying this atypical presentation remain currently unknown [28–31].

Clinically, osteomalacia manifests as diffuse skeletal pain, proximal myopathy, fractures of ribs/vertebrae/femoral neck, gait disturbance, and in severe cases, deformities; electrolyte imbalances may cause neuromuscular irritability [29, 32].

Radiographic signs of osteomalacia are discrete and appear in combination with other characteristic features of kidney bone disease. In osteomalacia, bone density is decreased, with loss of definition of the cortical bone and swelling of trabecular pattern. A rare but pathognomonic feature is the presence of Looser’s zones (i.e., Milkman’s fractures, pseudofractures), which are more common in the medial part of the femoral neck, pubic rami, ilii, scapulae, ribs, and acromion. Looser’s zones represent unmineralized osteoid and are seen as lucent symmetric lines perpendicular to the cortex; typically radiolucent lines do not extend across the whole width of the bone unless the fracture has occurred through it [33].

In osteomalacia, imaging techniques such as DEXA, vertebral morphometry, and MRI can support the diagnosis, although none are specific when used alone. DEXA typically shows reduced BMD in the spine, hips, or forearm; however, it cannot reliably distinguish osteomalacia from osteoporosis [29].

Subchondral insufficiency fractures may also be observed in weight-bearing joints, such as the knees. Vertebral morphometry may show biconcave or “codfish” vertebrae in chronic cases, although vertebral fractures are less frequent than in osteoporosis and early disease can present only with subtle vertebral height loss [34].

In our patient, due to the clinical worsening with progressive inability to ambulate independently, an MRI was performed following orthopedic recommendation, revealing diffuse bone marrow edema involving the patella, tibial plateaus, and femoral condyles. MRI may reveal bone marrow edema—which frequently occurs in various bone pathologies, appearing as high signal intensity on STIR or T2-weighted images—and bilateral.

In the patient described in this case report, several differential diagnoses were considered and excluded, including arthropathies, infection, osteonecrosis, and neoplastic lesions, based on clinical, laboratory, and imaging findings [35–37]. Although bone biopsy remains the gold standard, it was not performed. The combination of characteristic radiologic features (Looser’s zones, bone marrow edema), severe vitamin D deficiency, hypophosphatemia, elevated bone turnover markers, and the rapid clinical and imaging improvement after vitamin D supplementation supported osteomalacia as a major contributor. However, the overall clinical presentation was likely due to a combination of osteomalacia and persistent hyperparathyroid bone disease, both of which were addressed through treatment of vitamin D deficiency and post-transplant hyperparathyroidism.

In summary, bone marrow edema is not a primary pathology but rather a shared radiologic manifestation of underlying bone weakness and varied pathologies, highlighting the need to evaluate and address the root causes of reduced bone quality in affected patients (Table 3) [38, 39]. In our patient, the post‑transplant bone disease was not attributable to a single mechanism. The persistent hyperparathyroidism, already present before transplantation, continued to drive high‑turnover bone disease after engraftment. At the same time, the inappropriate suspension of native vitamin D supplementation and the introduction of glucocorticoids unveiled a significant pre‑existing vitamin D deficiency. This combination created the metabolic setting for osteomalacia to emerge, as confirmed by imaging and bone turnover markers. Therefore, the skeletal involvement in this case should be interpreted as the consequence of both persistent hyperparathyroidism and superimposed osteomalacia, rather than being explained by one mechanism alone. Due to the persistence of bone pain and in the absence of specific clinical or radiological signs of metabolic bone disease, skeletal scintigraphy was performed to rule out Paget’s disease. Skeletal scintigraphy with 99mTc-MDP revealed a focal area of radiotracer hyperaccumulation near the lateral wall of the right fourth rib, with no other significant areas of increased uptake suggestive of multifocal lesions or Paget’s disease .

**Table 3:** Types of osteomalacia

Type of osteomalacia	Causes	Main characteristics
Vitamin D deficiency osteomalacia	Decreased vitamin D production or absorption: inadequate sunlight, poor dietary intake, malabsorption disorders (e.g., celiac disease, Crohn’s disease)	Low calcium, low phosphate, secondary hyperparathyroidism
Osteomalacia due to alteration of vitamin D metabolism	Chronic kidney disease, nephrotic symptoms, pregnancy, liver diseases	Low 1,25(OH) vitamin D, seizures, bone deformities
X-linked hypophosphatemia	Renal phosphate wasting due to PHEX mutations or FGF23 excess	Serum phosphate is low; phosphate wasting in urine; bone deformities
Oncogenic osteomalacia (tumor-induced osteomalacia)	Secretion of FGF23 by mesenchymal tumors	Low serum phosphate; phosphate wasting;
Drug-induced osteomalacia	Anticonvulsants (phenytoin, phenobarbital), aluminum, excess bisphosphonate, isoniazid, rifampicin, ketoconazole, long term corticosteroid use	Serum abnormalities vary; depending on the interference with bone mineralization
Other causes (e.g., renal tubular acidosis, malabsorption, CKD)	Renal disorders, acid–base abnormalities, malabsorption, chronic disease, IV intravenous iron infusions	Serum abnormalities related to underlying disorders (calcium, phosphate, acid–base balance)

Osteomalacia is also a common cause of metabolic superscan, a characteristic bone scan pattern seen in various metabolic bone diseases [36]. This pattern is marked by diffuse and intense uptake of the radiotracer throughout the skeleton. Despite decreased bone mineralization in patients with osteomalacia, osteoid formation, calcium deposition, bone turnover, and PTH levels are increased and cause superscan patterns. Additionally, foci of increased uptake in pseudofractures (also known as Looser zones) can be observed in the femoral neck and scapula of patients [40].

An interesting finding in our case was the pattern of bone turnover markers before and after vitamin D treatment, reflecting coexistence of persistent hyperparathyroidism and vitamin D deficiency–related osteomalacia. Both conditions can elevate bone turnover markers, albeit with different mechanisms, and contribute to skeletal fragility; nevertheless, the rapid clinical and radiological improvement after vitamin D repletion suggests osteomalacia was the dominant factor in the acute presentation, while hyperparathyroidism persisted as a chronic background process. This distinction is important for the case interpretation and management in similar post-transplant scenarios.

DEXA and vertebral morphometry assess bone mass and detect subclinical fractures but cannot determine bone turnover, which is essential for identifying the disease phenotype and guiding therapy [41].

Emerging data suggest that bone turnover markers in CKD and KTRs might be used for the assessment of bone turnover, providing crucial insight into the dynamics of bone metabolism. To avoid bias related to kidney retention, in the setting of CKD, bone turnover markers that are not cleared by the kidneys, such as BALP, trimeric PINP1 and tartrate-resistant acid phosphatase-5b (TRAP-5b), must be considered and correlate more specifically with histological bone turnover in CKD compared to PTH alone. The use of PTH together with bone turnover markers improves the sensitivity and specificity of prediction models for both low and high bone turnover [41–43]. Monitoring their trend over time allowed us to evaluate therapeutic efficacy as well as patient compliance, helping to optimize therapy while minimizing the risk of adynamic bone disease [44, 45].

In our case, the clinical picture proved to be more complex than a classic vitamin D deficiency–related osteomalacia, as persistent hyperparathyroidism despite pharmacotherapy led to a simultaneous elevation of both bone formation and resorption markers and not to an isolated increase in BALP as typically seen in osteomalacia. Unfortunately, we were not able to determine PINP1 and TRAP-5b, as the assays were unavailable at that time. It can be postulated that the constellation of severe vitamin D deficiency, hypophosphatemia, imaging features such as Looser zones and bone marrow edema favored the diagnosis of osteomalacia rather than classic persistent hyperparathyroid bone disease. The underlying osteomalacia was likely already present in a subclinical form before transplantation and was unmasked and exacerbated by post‑transplant factors, including reduced sun exposure, steroid therapy, and discontinuation of vitamin D supplementation. The rapid clinical and radiological response to aggressive vitamin D replacement further supports osteomalacia as the predominant mechanism of bone fragility in this case.

The rationale for selecting solitary parathyroidectomy prior to transplantation remains unclear, particularly whether this choice was guided by preoperative findings or the identification of a single enlarged parathyroid gland, as the patient’s pre-transplant care was conducted at a different nephrology center.

In addition to severe vitamin D deficiency, the coexistence of a hypophosphatemia condition may have likely contributed to this patient’s onset of osteomalacia. These changes, including high FGF23, PTH, and increased urinary phosphate excretion (phosphaturia), typically resolve within a year post-transplant [14, 15]. Unfortunately, FGF23 was not measured; therefore, we cannot exclude that excess phosphatonin contributed to both vitamin D deficiency and hypophosphatemia.

The clinical presentation of osteomalacia is highly variable and often asymptomatic; however, in more severe cases, symptoms of hypocalcemia may appear along with marked muscle weakness and difficulty walking. The prevalence of osteomalacia in patients with advanced CKD and KTRs is probably much more frequent than expected. It is a fact that in KTRs bone biopsy studies, the presence of focal or generalized osteomalacia was surprisingly widespread even if the calcitriol levels were normal [20]. In the case described here, vitamin D deficiency was documented prior to transplantation and was likely longstanding. The effect of immunosuppressive therapy on this condition of severe vitamin D deficiency was evident, which might have contributed further, alongside the inappropriate discontinuation of cholecalciferol. The consensual presence of hypophosphatemia with multifactorial genesis (high PTH and FGF23 levels, vitamin D deficiency) feasibly played a further role in the onset of osteomalacia. It is important to underline that the observed improvement in biochemical and clinical parameters should not be attributed to vitamin D supplementation alone. In this case, the ongoing therapy with cinacalcet contributed significantly to the early control of hyperparathyroidism and hypercalcemia, while the later initiation of bisphosphonate treatment further supported the consolidation of bone metabolism and stabilization of BMD.

The evidence clearly indicates that vitamin D supplementation exerts a favorable effect on bone outcomes after kidney transplantation, likely mediated through improved control of hyperparathyroidism.

The target to be achieved in patients with CKD as well as KTRs remains a matter of debate. Our case suggests that preventing or correcting vitamin D deficiency in KTRs is likely a mandatory approach, given their unique pathophysiological and clinical profile and the heightened risk they face from the consequences of vitamin D deficiency. Given the vitamin D deficiency present in many transplant candidates, its identification and correction should be systematic and timely, in a clinical and pathophysiological context that inherently accentuates its severity. Similarly, the therapeutic regimen should not be limited to the simple correction of vitamin D deficiency but should be extended to all the complex alterations of mineral metabolism present in KTRs.

## Data Availability

No datasets were generated or analyzed during the current study.
